# Early-Onset Infection Caused by *Escherichia coli* Sequence Type 1193 in Late Preterm and Full-Term Neonates 

**DOI:** 10.3201/eid3001.230851

**Published:** 2024-01

**Authors:** Célie Malaure, Guillaume Geslain, André Birgy, Philippe Bidet, Isabelle Poilane, Margaux Allain, Mathilde Liberge, Nizar Khattat, Paola Sikias, Stéphane Bonacorsi

**Affiliations:** *Escherichia coli* National Reference Center, Robert-Debré University Hospital, Assistance Publique–Hôpitaux de Paris, Paris, France (C. Malaure, A. Birgy, P. Bidet, S. Bonacorsi);; Paediatric Intensive Care Unit, Robert-Debré University Hospital, Assistance Publique–Hôpitaux de Paris, Paris (G. Geslain);; Paris Cité University, Paris (G. Geslain, A. Birgy, P. Bidet, S. Bonacorsi);; Avicenne University Hospital, Assistance Publique–Hôpitaux de Paris, Bobigny, France (I. Poilane);; Louis Mourier University Hospital, Assistance Publique–Hôpitaux de Paris, Colombes, France (M. Allain);; Saint Louis University Hospital, Assistance Publique–Hôpitaux de Paris, Paris (M. Liberge);; Neonatal Care Unit, Robert-Debré University Hospital, Assistance Publique–Hôpitaux de Paris, Paris (N. Khattat);; Hôpital Privé d'Antony, Ramsay Santé, Antony, France (P. Sikias)

**Keywords:** neonatal sepsis, Escherichia coli, bacteria, antimicrobial resistance, whole-genome sequencing, meningitis/encephalitis, virulence, sequence type, ST1193, ST95, molecular epidemiology, France

## Abstract

Using whole-genome sequencing, we characterized *Escherichia coli* strains causing early-onset sepsis (EOS) in 32 neonatal cases from a 2019–2021 prospective multicenter study in France and compared them to *E. coli* strains collected from vaginal swab specimens from women in third-trimester gestation. We observed no major differences in phylogenetic groups or virulence profiles between the 2 collections. However, sequence type (ST) analysis showed the presence of 6/32 (19%) ST1193 strains causing EOS, the same frequency as in the highly virulent clonal group ST95. Three ST1193 strains caused meningitis, and 3 harbored extended-spectrum β-lactamase. No ST1193 strains were isolated from vaginal swab specimens. Emerging ST1193 appears to be highly prevalent, virulent, and antimicrobial resistant in neonates. However, the physiopathology of EOS caused by ST1193 has not yet been elucidated. Clinicians should be aware of the possible presence of *E. coli* ST1193 in prenatal and neonatal contexts and provide appropriate monitoring and treatment.

Extraintestinal pathogenic *Escherichia coli* and *Streptococcus agalactiae* are bacterial pathogens that commonly cause early-onset neonatal sepsis (EOS) in industrialized countries. EOS is confirmed by a blood or cerebrospinal fluid culture positive for the causative pathogen <72 hours after birth. EOS incidence is ≈1/1,000 live births (*1*,*2*); 10% of cases are complicated by meningitis, which can lead to neurologic sequelae in up to 50% and death in 10% of cases in industrialized countries (*3*). 

EOS caused by *S. agalactiae* can be prevented by peripartum antimicrobial prophylaxis but not EOS caused by *E. coli*. *E. coli* strains that cause neonatal meningitis have been well characterized, but *E. coli* strains that cause EOS less so (*4*,*5*). Neonatal meningitis *E. coli* strains belong mainly to phylogenetic group B2/sequence type complex (STc) 95 (*6*) and are frequently O18:K1, O1:K1, O83:K1, or O45_S88_:K1 serotypes (*7*,*8*). Most STc95 strains are distributed worldwide and still largely susceptible to antimicrobials (*9*). However, other strains that can cause EOS, notably in preterm neonates, might be resistant to probabilistic antimicrobial therapy. In a recent study in Israel (*10*), maternal carriage rates of extended-spectrum β-lactamase (ESBL)–producing *E. coli* were 17.5% for mothers and 12.9% for preterm neonates; in China, ESBL accounted for up to 48% of *E. coli* infections in neonates (*11*).

Characterizing *E. coli* strains that cause EOS would constitute a critical first step towards better understanding the pathophysiology of this condition and developing potential preventive strategies. We conducted a prospective study covering a large area in France to estimate annual incidence and pathogen distribution of EOS in neonates born at ≥34 weeks of gestation during 2019–2021 (*12*). In total, we recorded 107 cases of bacteremia including 35 caused by *E. coli*, 15 (incidence 0.89/1,000 births) in late-preterm and 20 (0.06/1,000 births) in full-term infants. We prospectively recorded data on maternal and infant demographics, maternal antimicrobial therapy, peripartum antimicrobial prophylaxis, and outcomes (*12*). We aimed to use whole-genome sequencing (WGS) to characterize *E. coli* strains that caused EOS in cases from this prospective study and stratify results according to these data. In addition, we determined to compare those strains to *E. coli* strains obtained from cultures from vaginal swabs collected to screen for *S. agalactiae* carriage at 34–38 weeks of gestation from woman with newborns who had no history of EOS. The ethics committee institutional review board (Ramsay Santé Recherche & Enseignement, IRB00010835) authorized the study (*12*). 

## Methods

### Bacterial Strains

We recorded 35 cases of EOS caused by *E. coli* during a prospective study in 81 maternity wards of the Ile de France area during 2019–2021 (*12*). Thirty-two *E. coli* isolates were sent to the National Reference Center in Robert-Debré Hospital to be further characterized. For comparison with the isolates from the Ile de France study, we included 50 *E. coli* isolates obtained from cultures from vaginal swabs collected from 4 maternity wards to screen pregnant woman for *S. agalactiae* carriage at 34–38 weeks of gestation. We found healthy vaginal carriage (HVC) among all; that is, none of the infants of the pregnant women from the *S. agalactiae* screening developed EOS caused by *E. coli*. 

### Antimicrobial Susceptibility Testing and Phenotypic Characterization 

We determined antimicrobial susceptibility of the *E. coli* strains using disk diffusion on Mueller-Hinton agar plates (bioMérieux, https://www.biomerieux.com), as recommended by Comité de l’Antibiogramme de la Société Française de Microbiologie (https://www.sfm-microbiologie.org) guidelines. We defined ESBL production by synergy between clavulanic acid and >1 extended-spectrum cephalosporin or aztreonam. 

### Molecular Characterization

We performed WGS on 82 isolates, 32 described elsewhere (*12*) and 50 from the HVC/*S. agalactiae* screening. We extracted bacterial genomic DNA using the DNeasy UltraClean Microbial Kit (QIAGEN, https://www.qiagen.com) and prepared libraries using Nextera Flex/DNA Prep library kits (Illumina, https://www.illumina.com) as specified by the manufacturers. We performed sequencing using 2 × 150 bp MiniSeq technology (Illumina) and assembled models using SPAdes (https://github.com/ablab/spades). We estimated quality of sequencing data using standard metrics, including N50 and mean coverage (Appendix). We determined phylogenetic groups, serotypes, fimH type, sequence type (ST), and STcs (which regroup all STs of <1 allele difference), whole-genome multilocus sequence typing (MLST), and hierarchical clustering of core genome MLST using Enterobase (https://enterobase.warwick.ac.uk) (*13*). We used the Center for Genomic Epidemiology website (https://genomicepidemiology.org) to search for resistance and virulence genes. We also used a local BLAST with a collection of virulence genes as described elsewhere (*14*). We used Fisher exact analysis for statistical comparisons among groups. 

## Results

### Bacterial Collection and Demographic and Clinical Features of Patients

We studied 82 *E. coli* isolates. Birth locations of the neonates within Ile de France were diverse (30 different locations among 32 EOS case-patients). Babies were delivered at full term (≥37 weeks of gestation) in 59% (19/32) and preterm (<37 weeks of gestation) in 41% (13/32) of cases. In 6 (31%) cases from the full-term group and 7 cases (54%) from the preterm group, mothers received antimicrobial treatment <3 days before labor. We observed 6 cases of meningitis, 3 each from the full-term and preterm neonate groups (Table 1). 

**Table 1:** Characteristics of mothers and their newborns with early-onset sepsis caused by *Escherichia coli* strains, France*

Case ID	Birth city†	Pregnancy term, wk	Prepartum antimicrobial‡	Meningitis§	Serotypes¶	FimH type	ST (STc)#	ESBL
APIMF52	Pontoise	37	–	–	O18:K1	fimH15	95	–
APIMF53	Antony	38	–	–	O13:K1	fimH21	357	–
APIMF54	Paris (Robert Debre)	35	+	–	O7:K5	fimH27	93 (168)	–
APIMF56	Orsay	41	+	–	O84	fimH38	2040	–
APIMF57	Clamart	40	+	–	O8	fimH32	58 (155)	–
APIMF58	Clichy	36	+	–	O18:K5	fimH27	14	–
APIMF59	Paris (Necker)	36	–	–	O25:K5	fimH30	131	+
APIMF60	Paris (Clinique Bleuets)	37	+	+	O75:K1	fimH64	1193 (14)	+
APIMF63	Quincy Sous Senart	39	–	–	O2:K1	fimH34	10	–
APIMF67	Mantes-La-Jolie	39	–	–	O18:K1	fimH107	95	–
APIMF68	Paris (Pitie)	40	–	–	O2:K1	fimH27	95	–
APIMF69	Saint Cloud	35	–	–	O18:K1	fimH15	95	–
APIMF70	Saint Denis	40	–	–	O25:K1	fimH41	59	–
APIMF71	Paris (Trousseau)	37	–	–	O7:K1	fimH1	80 (568)	–
APIMF72	Aulnay	36	–	–	O1:K1	fimH41	95	–
APIMF73	Villeneuve Saint-Georges	34	+	–	O75:K1	fimH64	1193 (14)	+
APIMF76	Colombes	41	–	–	O75:K1	fimH64	1193 (14)	–
APIMF77	Nogent Sur Marne	41	–	–	O17/O77:K52	Unknown	394	–
APIMF78	Coulommiers	41	–	+	O75:K1	fimH64	1193 (14)	–
APIMF79	Longjumeau	38	–	–	O6:K23	fimH103	73	–
APIMF80	Eaubonne	38	–	–	O4:K96	fimH5	12	–
APIMF81	Jossigny	34	–	–	O75:K5	fimH27	14	–
APIMF82	Meaux	34	+	–	O25:K5	fimH28	2279 (131)	–
APIMF83	Saint-Maurice	39	+	–	O8	fimH29	58 (155)	–
APIMF84	Creteil	39	+	–	O15:K96	fimH30	69	–
APIMF85	Arpajon	41	–	–	O4:Ku	fimH8–like	12	–
APIMF87	Paris (Trousseau)	35	–	+	O7:K1	fimH44	62	–
APIMF88	Marne La Vallee	36	+	+	O7:K1	fimH44	62	–
APIMF89	Creteil	36	+	+	O75:K1	fimH64	1193 (14)	+
APIMF90	Levallois Perret	36	+	–	O75:K1	fimH64	1193 (14)	–
APIMF91	Melun	34	–	–	O1:K1	fimH41	59	–
APIMF92	Beaumont Sur Oise	40	+	+	O18:K1	fimH15	95	–

*ESBL, extended spectrum β-lactamase; FimH, type 1 fimbriae D-mannose specific adhesin; ID, identification;Ku, unknown serogroup capsular; +, positive for condition; –, negative for condition. †For Paris birthplaces, name of hospital where born is indicated in parentheses. ‡Amoxicillin was the only antimicrobial prescribed within 3 days before labor. §Early onset neonatal meningitis. ¶O antigen predicted with SerotypeFinder 2.0 and serogroup capsular predicted by the Center for Genomic Epidemiology (https://genomicepidemiology.org). #Sequence type complex (STc) indicated in parentheses if different from ST number.

### Diversity and Phylogenetics of EOS and HVC *E. coli* Strain Collections

**Figure 1:**
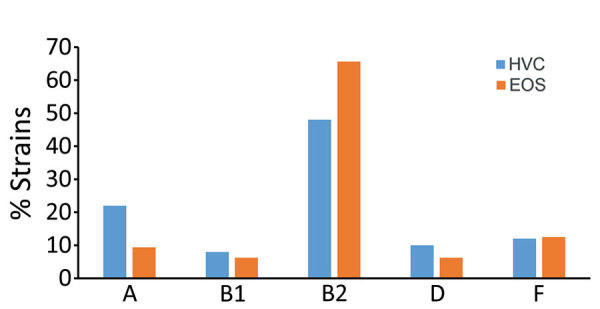
Phylogroup distribution among 32 EOS *Escherichia coli* strains from neonates and 50 HVC strains, France. No significant difference was observed in each group. EOS, early-onset neonatal sepsis; HVC, healthy vaginal carriage.

**Figure 2:**
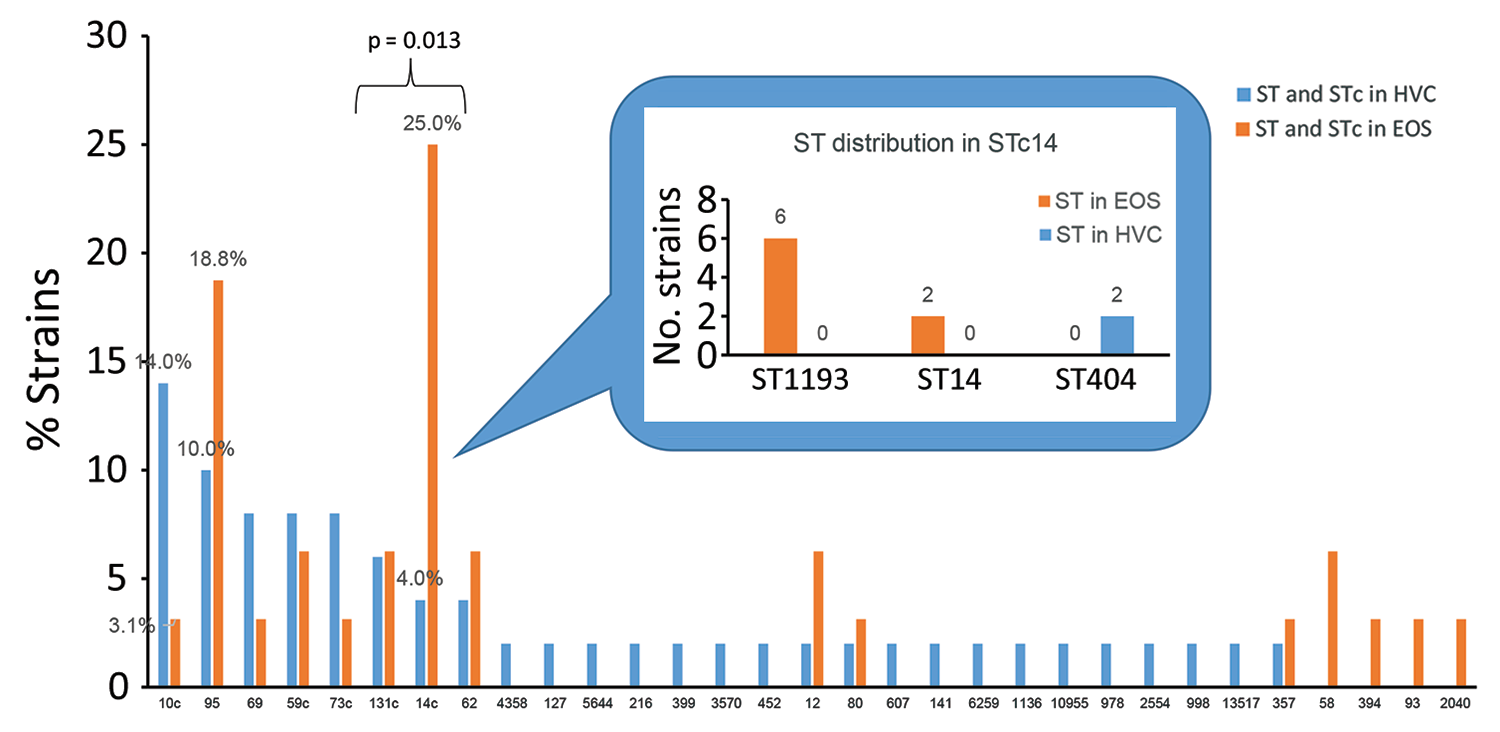
ST and STc distributions of EOS neonate and HVC *Escherichia coli* strains, France. STc14 distribution is detailed. STc10 includes ST10, ST13795, ST6826, and ST13957; STc59 includes ST59, ST415, and ST13796; STc11 includes ST73 and ST355; STc131 includes ST131 and ST2279. EOS, early-onset neonatal sepsis; HVC, healthy vaginal carriage; ST, sequence type; STc, ST complex.

Five of 7 major *E. coli* phylogroups—A, B1, B2, D, and F, but not C or E—were represented in similar proportions in both the Ile de France study and vaginal swab collections (p>0.05). The exceptions to this trend were phylogroup A being more common in vaginal swab (22%) than EOS (9.4%) isolates and group B2 more common in EOS (65.6%) than vaginal swab (48%) isolates (Figure 1). Among the 3 most frequent ST/STc variants in our study, STc10 (phylogroup A) was present in more HVC strains, whereas ST95 and STc14 (phylogroup B2) were more common in EOS strains. The imbalance was striking for STc14, which was present in 25% of EOS strains but only 4% of HVC strains (p = 0.01) (Figure 2). STc14 isolates included 6 ST1193, 2 ST14, and 2 ST404. Of note, the 6 ST1193 isolates were found exclusively in the EOS collection. 

### Virulence and Antimicrobial Resistance

**Figure 3:**
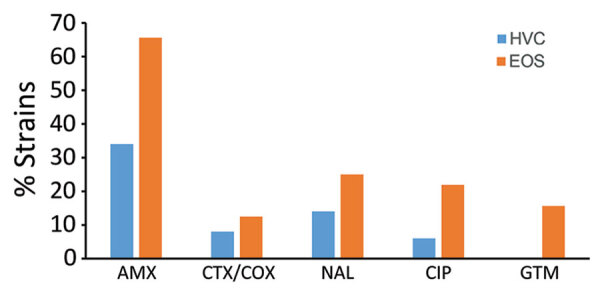
Antibiotic resistance rates among EOS neonate and HVC *Escherichia coli* strains, France. AMX, amoxicillin; CIP, ciprofloxacin; CTX/COX, cefotaxime/ceftriaxone; EOS, early-onset neonatal sepsis; GTM, gentamicin; HVC, healthy vaginal carriage; NAL, nalidixic acid.

We observed no significant difference in distribution of virulence factors between the EOS and HVC strain collections, except for genes encoding the K1 capsule, which were present significantly more in the EOS collection (Appendix Table 1). In contrast, antimicrobial resistance differed markedly between collections (Figure 3). Aminopenicillin resistance was ≈2 times higher among EOS (65.6%) than HVC (34%) collection strains (p = 0.007); ESBL was present in 12.5% of EOS and 8% of HVC strains (p>0.05). Resistance to fluoroquinolone and gentamicin were also more common among EOS strains (Figure 3). 

**Figure 4:**
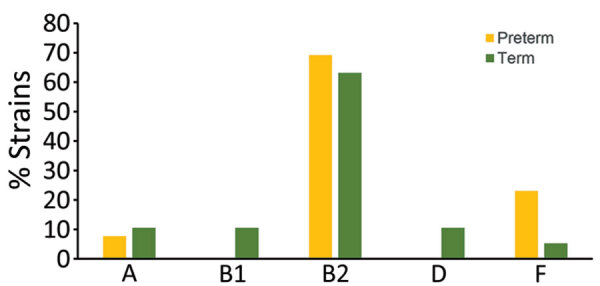
Phylogroup distributions according to birth term among 32 neonates with early-onset sepsis, France.

**Figure 5:**
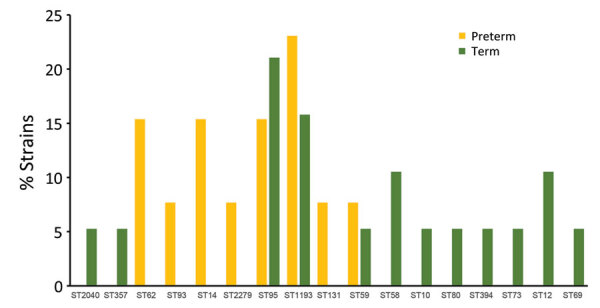
ST distributions according to birth term among 32 neonates with early-onset neonatal sepsis. ST, sequence type.

**Figure 6:**
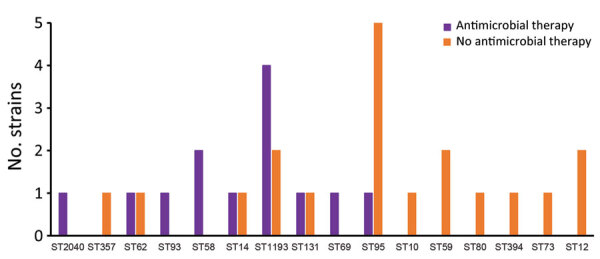
Distribution of maternal antimicrobial therapy within 3 days before delivery according to *Escherichia coli* ST among 32 neonates with early-onset neonatal sepsis, France. ST, sequence type.

We examined the distribution of phylogenetic groups and ST/STc frequency among EOS strains stratified by gestational term of newborns. Differences in rates of B2 phylogroup strains in the 2 subpopulations (69% in preterm, 63% in full-term neonates) were not statistically significant (Figure 4). STc14 (ST14/ST1193) was >2 times as frequent in the preterm (38.5%) as the full-term subpopulation (15.8%), but the difference was not statistically significant (p>0.05). Distribution of ST95, the second most frequent ST, was similar between preterm (15.4%) and full-term (21.1%) subpopulations (Figure 5). There were more mothers with STc14 *E. coli* isolates (5/13 [38.5%]) among those who received antimicrobial therapy <3 days before delivery than those who did not (3/19 [15.8%]) (p>0.05) (Figure 6). In contrast, there were fewer ST95 isolates among mothers receiving prenatal antimicrobial therapy (1/13 [7.69%]) than those receiving no therapy (5/19 [26.32%]) (p>0.05). 

### Main Features of EOS Caused by ST1193 *E. coli* and Characterization of Isolates

All 6 ST1193 EOS strains were isolated from different maternity hospitals. Half (3/6) of neonates with ST1193 EOS were born at full term. Three neonates had meningitis, 2 full-term and 1 preterm. Four (67%) mothers with ST1193 strains received prenatal antimicrobial therapy compared with 9 (35%) for the non-ST1193 strains (p>0.05) (Table 1; Figure 6). All strains were resistant to fluoroquinolones, 3 were resistant to azithromycin, and 3 others harbored an ESBL phenotype (Table 2). All strains were lactose nonfermenters (data not shown). 

**Table 2:** Characteristics of newborns with early-onset sepsis caused by ST1193 *Escherichia coli* and genetic and phenotypic features of the isolates*

APIMF case ID	EOS clinical features			Genetic and phenotypic characteristic of *E. coli* strains ST1193					
	Term, wk†	Antimicrobial prepartum‡		Virulence genes§	Antimicrobial resistance genes§	Phenotypic AMR	HC10¶	HC20¶	rMLST#
60**	37	Amoxicillin		*aslA*,* chuA*,* csgA*,* fdeC*,* fimH*,* fyuA*,* gad*,* iha*,* irp2*,* iucC*,* iutA*,* kpsE*,* kpsMII_K1*,* neuC*,* nlpI*,* ompT*,* papA_F43*,* sat*,* senB*,* shiA*,* sitA*,* terC*,* tia*,* usp*,* vat*,* yehA*,* yehB*,* yehC*,* yehD*,* yfcV*	*dfrA17*,* aph(6)-Id*,* aac(6')-Ib-cr*,* aac(3)-IIa*, *aph(3”)-Ib*,* sul2*,* catB3*,*mph(A)*,* sitABCD*,* tet(B)*,* bla*_CTX-M-15_,* bla*_TEM-1B_,* bla*_OXA-1_	AMX, CTX, CIP, SXT, GTM, AZI, TET, CMP, ESBL	132,558	571	33,503	
73	34	Amoxicillin		*aslA*,* chuA*,* csgA*,* fdeC*,* fimH*,* fyuA*,* gad*,* iha*,* irp2*,* iucC*,* iutA*,* kpsE*,* kpsMII_K1*,* neuC*,* nlpI*,* ompT*,* papA_F43*,* sat*,* senB*,* sitA*,* terC*,* usp*,* vat*,* yehA*,* yehB*,* yehC*,* yehD*,* yfcV*	*catB3*,* tet(B)*,* bla*_CTX-M-15_,* bla*_OXA-1_, *aph(6)-Id*,* aac(3)-IIa*,* aph(3”)-Ib*,* aac(6')-Ib-cr*,* sul2*,* aac(6')-Ib-cr*,* qnrB19*,* dfrA17*	AMX, CTX, CIP, SXT, GTM, TET, ESBL	148,092	571	33,503	
76	41	None		*aslA*,* ccl*,* chuA*,* clbB*,* csgA*,* fdeC*,* fimH*,* fyuA*,* gad*,* iha*,* irp2*,* iucC*,* iutA*,* kpsE*,* kpsMII_K1*,* neuC*,* nlpI*,* ompT*,* papA_F43*,* sat*,* senB*,* sitA*,* terC*,* usp*,* vat*,* yehA*,* yehB*,* yehC*,* yehD*,* yfcV*	None	CIP	227,323	571	1,674	
78**	41	None		*AslA*,* chuA*,* csgA*,* fdeC*,* fimH*,* fyuA*,* gad*,* iha*,* irp2*,* iucC*,* iutA*,* kpsE*,* kpsMII_K1*,* neuC*,* nlpI*,* ompT*,* papA_F43*,* sat*,* senB*,* sitA*,* terC*,* usp*,* vat*,* yehA*,* yehB*,* yehC*,* yehD*,* yfcV*	*aadA5*,* dfrA17*,* mph(A)*	CIP, AZI, SXT	11,740	571	1,674	
89**	36	Amoxicillin/ gentamicin		*AslA*,* astA*,* chuA*,* colE7*,* csgA*,* fdeC*,* fimH*,* fyuA*,* gad*,* iha*,* irp2*,* iucC*,* iutA*,* kpsE*,* kpsMII_K1*,* neuC*,* nlpI*,* ompT*,* papA_F43*,* sat*,* senB*,* shiA*,* sitA*,* terC*,* tia*,* usp*,* vat*,* yehA*,* yehB*,* yehC*,* yehD*,* yfcV*	*aac(3)-IIa*,* aac(6')Ib-cr*,* bla*_CTX-M-15_, *bla*_OXA-1_, *catB3*	AMX, CTX, CIP, GTM, CMP, ESBL	4,073	571	33,503	
90	36	Amoxicillin		*AslA*,* chuA*,* csgA*,* fdeC*,* fimH*,* fyuA*,* gad*,* iha*,* irp2*,* iucC*,* iutA*,* kpsE*,* kpsMII_K1*,* neuC*,* nlpI*,* ompT*,* papA_F43*,* sat*,* senB*,* sitA*,* terC*,* usp*,* vat*,* yehA*,* yehB*,* yehC*,* yehD*,* yfcV*	*bla*_TEM-1B_, *dfrA17*,* mph(A)*,* aph(6)-Id*,* aph(3”)-Ib*,* sul2*,* tet(B)*	AMX, CIP, SXT, AZI, TET	227,336	571	New	

*AMR, antimicrobial resistance; AMX, amoxicillin; AZI, azithromycin; cgMLST, core genome multilocus sequence typing; CIP, ciprofloxacin; CMP, chloramphenicol; CTX, cefotaxime; EOS, early-onset neonatal sepsis; ESBL, extended spectrum β-lactamase; GTM, gentamicin; HC, human chromosome; ID, identification; rMLST, ribosomal multilocus sequence typing; ST, sequence type; SXT, trimethoprim/sulfamethoxazole; TET, tetracycline. †Term of pregnancy at birth in weeks. ‡Antimicrobial treatment within 3 days before labor began. §Search for antimicrobial resistance genes and replicon/plasmid sequence types run using Center for Genomic Epidemiology website (https://genomicepidemiology.org) and local BLAST. ¶Hierarchical clustering of distances between genomes calculated using 10 (HC10) or 20 (HC20) shared core genome MLST alleles and genomes linked on a single-linkage clustering criteria. #Approach that indexes variation of the 53 genes encoding the bacterial ribosome protein subunits. **Early-onset neonatal sepsis with meningitis.

We assessed presence of putative virulence factors among the 6 ST1193 isolates (Table 2; Appendix Table 2) and identified presence of factors with a significant difference (p<0.05) among ST1193 compared with non-ST1193 strains: adherence protein *Iha*, colicin Ia immunity protein *Imm*, major pilin subunit *PapA_F43*, plasmid-encoded enterotoxin *SenB*, serine protease *Sat*, vacuolating autotransporter toxin *Vat*, and Type 1 fimbrin D-mannose specific adhesion 64. 

Three strains harbored ESBL phenotypes contained the β-lactamase–encoding genes *bla*_CTX-M-15_ and *bla*_OXA-1_ associated with the *aac(6')-Ib-cr* genes, and 3/6 strains harbored the *mph(A)* gene (macrolide 2′-phosphotransferase), which inactivates macrolides, reinforcing observed phenotypic resistance to azithromycin. None of the non-ST1193 strains carried that gene. One strain was resistant only to fluoroquinolones (Table 2). All strains had different hierarchical cluster 10 (HC10) but the same HC20 (571), whereas ribosomal MLST (rMLST) split the strains into 2 main populations: rMLST 33503, which regroups the 3 ESBL-producing strains, and rMLST 1674, which contains 2 less-resistant isolates. 

## Discussion

In our study, we used WGS to characterize *E. coli* strains causing EOS from a prospective multicenter study in France (*12*) and compared them to *E. coli* strains obtained from vaginal samples from pregnant women at 34–38 weeks of gestation. Although we observed no major differences between the EOS study and vaginal sample collections in distribution of phylogroups or virulence factors except the K1 antigen, we identified emerging ST1193 strains as major causes of EOS. Three isolates of the ST1193 clonal group caused meningitis, and half harbored an ESBL. *E. coli* ST1193 thus appears to be the most virulent and antimicrobial-resistant *E. coli* group that causes EOS. 

Among major phylogroups, B2 and, to a lesser extent, D are associated with extraintestinal infections, whereas A and B1 are most associated with commensal strains or intestinal infections (*15*). We also observed predominance of B2 strains in our EOS population, regardless of the term of birth of the newborns. Although the proportion of phylogroup A strains was higher in the HVC than the EOS population, B2 strains largely predominated in the HVC collection, as reported in previous studies (*16*,*17*). However, sequence typing enabled a finer comparison between the 2 collections. Among the HVC strains, phylogroup A/STc10 (ST10, ST13795, ST6826, and ST13957) was predominant but was rarely observed among the EOS patients, in which ST95 and STc14 (notably ST14 and ST1193) were largely predominant. The high frequency of ST95 was expected because of its virulence in neonates, notably those with neonatal meningitis, which is well known worldwide (*6*,*18*). Of note, ST95 was second most common among HVC strains, suggesting its capacity to colonize the vagina, at least temporarily. Five of 6 mothers with EOS caused by ST95 received no prepartum antimicrobials. In contrast, ST14 and ST1193 strains were frequently associated with women receiving prepartum antimicrobials (5/8), and those strains were not present among HVC patients, suggesting the vaginal environment might inhibit the presence of ST14 and ST1193 strains. Of note, that STc14 but not ST95 was more prevalent among preterm neonates with EOS and 3/6 infections caused by ST1193 strains occurred in preterm newborns. It might be that ST1193 strains are less virulent than ST95 strains commonly found in full-term neonates. However, almost all women with preterm newborns received antimicrobial drugs, which might favor the selection of resistant strains, such as ST1193. 

ST1193 was identified within STc14 approximately 25 years ago; its prevalence in extraintestinal infections could become a public health burden (*19*–*21*). One study observed an increased rate of ST1193 causing bloodstream infections, mostly in elderly patients in Canada during 2016–2018 (*22*). In an analysis of the population structure of 218 ESBL–producing *E. coli* in urinary tract infections in febrile children in France during 2014–2017, we noted prevalence of ST1193 rose from 0% to 9% (*23*). Large epidemiologic studies of ST1193 prevalence in neonatal infection have only recently been conducted. In 2 studies, ST1193 was shown to be a major cause of neonatal sepsis; however, because the definition of EOS in those studies differed from ours, data are difficult to compare (*11*,*24*). The finding of a worrying percentage of ST1193 among EOS patients (19%) in our study population indicates that in the future that ST should be closely monitored using microbiologic detection. 

One epidemiologic study of intracranial infections in neonates caused by *E. coli* (*25*) found ST1193 to be the most prevalent ST (28%). All 8 ST1193 isolates caused late-onset infections, although none caused EOS. Only 1 recent case of early-onset meningitis caused by *E. coli* ST1193 has been reported, but cases of meningitis caused by ST1193 occurring >72 hours after birth were described in another study (*24*,*26*). The recent case occurred in a late-preterm neonate with a history of prolonged rupture of the membrane with prepartum and peripartum antimicrobial drugs administered, as in most of our cases. 

Given that 3/6 ST1193 strains caused neonatal meningitis, such strains were shown to have high invasive disease potential in newborns. Several virulence factors and genetic determinants have been shown to be involved in the pathophysiology of neonatal meningitis, such as capsule K1, siderophore salmochelin, plasmid *pS88*, and invasin *IbeA* (*27*). Of note, among these determinants, only the K1 capsule was present in the ST1193 strains. Several virulence factors (Iha, Imm, plasmid-encoded enterotoxin SenB, Sat) were present in all ST1193 strains, with a significant p value (p<0.05) compared with non-ST1193 (Appendix Table 2) strains, and were present in >85% of ST1193 strains in the large collection of 1 study (*28*). Therefore, without in vivo study, it is difficult to determine the specific roles of these key factors in the invasiveness of ST1193 in cerebrospinal fluid. 

Except for consistency of fluoroquinolone resistance and carrying the fimH64 allele, which characterized all ST1193 *E. coli* strains described in previous studies, multiple plasmid-borne resistance genes have been reported but are inconsistently associated with ST1193 (*19*,*28*,*29*). No isolates harbored the same phenotypic antimicrobial resistance pattern, highlighting their diversity. The co-occurrence of *bla*_CTX-M-15_/*bla*_OXA-1_/*aac(6*′*)-Ib-cr*, which we observed in 3/6 of EOS strains, has been frequently described, initially in ST131 but also more recently in emerging lineages of ST1193 (*30*). Half of our strains, similar to findings from other studies (*28*), carried the *mph*A resistance gene and had a high azithromycin MIC (>32 mg/L) (data not shown), which might have contributed to the emergence of ST1193 given that azithromycin is among the most-prescribed antimicrobial drugs worldwide among adult outpatients (*31*). 

As of May 2023, sequences of 2,031 *E. coli* ST1193 strains from all over the world are available in Enterobase (*13*). Of those, 80% belong to HC20 571, as did our strains, and most (82%) harbor rMLST 1674, whereas rMLST 33503 is found in only 8%. Hierarchical clustering analysis did not suggest the presence of a particular clone in our collection. Distribution of rMLSTs was notably different: half of our ST1193 strains belonged to rMLST 33503. Whether this subgroup is emerging or has specific invasive disease potential in neonates has yet to be determined.

Among its strengths, our prospective epidemiologic study, conducted in a large area of France, estimated annual incidence and pathogen distribution in EOS patients (*12*) and documented the unique molecular and phenotypic characteristics of the strains in our study. We were limited by the small number of patients; results, especially implication of ST1193 in infections in very preterm neonates, need to be confirmed in larger study populations. 

In conclusion, our findings suggest that ST1193 is emerging as a major *E. coli* pathogen that can cause EOS and early-onset neonatal meningitis in full-term and late-preterm newborns and might surpass ST95 in incidence and causing illness because of its potential virulence combined with its resistance to multiple antimicrobials. Pediatricians and microbiologists should be aware of the public health threat from *E. coli* ST1193 and the benefits of prepartum/peripartum EOS treatment with effective antimicrobials. Isolating ST1193 *E. coli* strains in the neonatal context (from mother, newborn, or both) will require careful, sustained clinical monitoring of newborns. It might also require implementing measures to limit spread, especially in neonatal wards. On the basis of microbiologic evidence, ST1193 should be suspected when 3 properties are all present: high resistance to ciprofloxacin, K1 capsule, and non–lactose-fermenting colonies, each of which can easily be tested for in a microbiology laboratory. Further studies should help to define the genetic determinants of ST1193 virulence in neonates and confirm and subsequently explain its inability or weak ability to colonize the vagina. Clinicians need to be aware of the possible presence of *E. coli* ST1193 in prenatal and neonatal contexts and provide appropriate monitoring and treatment. 

Appendix Additional information about early-onset infection caused by *Escherichia coli* ST1193 in late preterm and full-term neonates.

## References

[1] Shane AL, Sánchez PJ, Stoll BJ. Neonatal sepsis. Lancet. 2017;390:1770–80. 10.1016/S0140-6736(17)31002-4 28434651

[2] Stoll BJ, Puopolo KM, Hansen NI, Sánchez PJ, Bell EF, Carlo WA, et al.; Eunice Kennedy Shriver National Institute of Child Health and Human Development Neonatal Research Network. Early-onset neonatal sepsis 2015 to 2017, the rise of *Escherichia coli*, and the need for novel prevention strategies. JAMA Pediatr. 2020;174:e200593. 10.1001/jamapediatrics.2020.0593 32364598 PMC7199167

[3] Gaschignard J, Levy C, Romain O, Cohen R, Bingen E, Aujard Y, et al. Neonatal bacterial meningitis: 444 cases in 7 years. Pediatr Infect Dis J. 2011;30:212–7. 10.1097/INF.0b013e3181fab1e7 21416693

[4] Joshi NS, Huynh K, Lu T, Lee HC, Frymoyer A. Epidemiology and trends in neonatal early onset sepsis in California, 2010-2017. J Perinatol. 2022;42:940–6. 10.1038/s41372-022-01393-7 35469043

[5] Miselli F, Cuoghi Costantini R, Creti R, Sforza F, Fanaro S, Ciccia M, et al. *Escherichia coli* is overtaking group B *Streptococcus* in early-onset neonatal sepsis. Microorganisms. 2022;10:10. 10.3390/microorganisms10101878 36296155 PMC9607315

[6] Basmaci R, Bonacorsi S, Bidet P, Biran V, Aujard Y, Bingen E, et al. *Escherichia coli* meningitis features in 325 children from 2001 to 2013 in France. Clin Infect Dis. 2015;61:779–86. 10.1093/cid/civ367 25944342

[7] Bonacorsi S, Clermont O, Houdouin V, Cordevant C, Brahimi N, Marecat A, et al. Molecular analysis and experimental virulence of French and North American *Escherichia coli* neonatal meningitis isolates: identification of a new virulent clone. J Infect Dis. 2003;187:1895–906. 10.1086/375347 12792866

[8] Peigne C, Bidet P, Mahjoub-Messai F, Plainvert C, Barbe V, Médigue C, et al. The plasmid of *Escherichia coli* strain S88 (O45:K1:H7) that causes neonatal meningitis is closely related to avian pathogenic *E. coli* plasmids and is associated with high-level bacteremia in a neonatal rat meningitis model. Infect Immun. 2009;77:2272–84. 10.1128/IAI.01333-08 19307211 PMC2687354

[9] Riley LW. Pandemic lineages of extraintestinal pathogenic *Escherichia coli.* Clin Microbiol Infect. 2014;20:380–90. 10.1111/1469-0691.12646 24766445

[10] Frank Wolf M, Abu Shqara R, Naskovica K, Zilberfarb IA, Sgayer I, Glikman D, et al. Vertical transmission of extended-spectrum, beta-lactamase-producing *Enterobacteriaceae* during preterm delivery: a prospective study. Microorganisms. 2021;9:506. 10.3390/microorganisms9030506 33673648 PMC7997221

[11] Gu S, Lai J, Kang W, Li Y, Zhu X, Ji T, et al. Drug resistance characteristics and molecular typing of *Escherichia coli* isolates from neonates in class A tertiary hospitals: A multicentre study across China. J Infect. 2022;85:499–506. 10.1016/j.jinf.2022.09.014 36245138

[12] Sikias P, Biran V, Foix-L’Hélias L, Plainvert C, Boileau P, Bonacorsi S; EOS study group. Early-onset neonatal sepsis in the Paris area: a population-based surveillance study from 2019 to 2021. Arch Dis Child Fetal Neonatal Ed. 2023;108:114–20. 10.1136/archdischild-2022-324080 35902218 PMC9985718

[13] Zhou Z, Alikhan N-F, Mohamed K, Fan Y, Achtman M; Agama Study Group. The EnteroBase user’s guide, with case studies on *Salmonella* transmissions, *Yersinia pestis* phylogeny, and *Escherichia* core genomic diversity. Genome Res. 2020;30:138–52. 10.1101/gr.251678.119 31809257 PMC6961584

[14] Cointe A, Birgy A, Mariani-Kurkdjian P, Liguori S, Courroux C, Blanco J, et al. Emerging multidrug-resistant hybrid pathotype Shiga toxin–producing *Escherichia coli* O80 and related strains of clonal complex 165, Europe. Emerg Infect Dis. 2018;24:2262–9. 10.3201/eid2412.180272 30457551 PMC6256387

[15] Denamur E, Clermont O, Bonacorsi S, Gordon D. The population genetics of pathogenic *Escherichia coli.* Nat Rev Microbiol. 2021;19:37–54. 10.1038/s41579-020-0416-x 32826992

[16] Sáez-López E, Cossa A, Benmessaoud R, Madrid L, Moraleda C, Villanueva S, et al. Characterization of vaginal *Escherichia coli* isolated from pregnant women in two different African sites. PLoS One. 2016;11:e0158695. 10.1371/journal.pone.0158695 27387665 PMC4936694

[17] Watt S, Lanotte P, Mereghetti L, Moulin-Schouleur M, Picard B, Quentin R. *Escherichia coli* strains from pregnant women and neonates: intraspecies genetic distribution and prevalence of virulence factors. J Clin Microbiol. 2003;41:1929–35. 10.1128/JCM.41.5.1929-1935.2003 12734229 PMC154741

[18] Johnson TJ, Wannemuehler Y, Johnson SJ, Stell AL, Doetkott C, Johnson JR, et al. Comparison of extraintestinal pathogenic *Escherichia coli* strains from human and avian sources reveals a mixed subset representing potential zoonotic pathogens. Appl Environ Microbiol. 2008;74:7043–50. 10.1128/AEM.01395-08 18820066 PMC2583479

[19] Pitout JDD, Peirano G, Chen L, DeVinney R, Matsumura Y. *Escherichia coli* ST1193: Following in the Footsteps of *E. coli* ST131. Antimicrob Agents Chemother. 2022;66:e0051122. 10.1128/aac.00511-22 35658504 PMC9295538

[20] Crémet L, Caroff N, Giraudeau C, Reynaud A, Caillon J, Corvec S. Detection of clonally related *Escherichia coli* isolates producing different CMY β-lactamases from a cystic fibrosis patient. J Antimicrob Chemother. 2013;68:1032–5. 10.1093/jac/dks520 23302581

[21] Platell JL, Trott DJ, Johnson JR, Heisig P, Heisig A, Clabots CR, et al. Prominence of an O75 clonal group (clonal complex 14) among non-ST131 fluoroquinolone-resistant *Escherichia coli* causing extraintestinal infections in humans and dogs in Australia. Antimicrob Agents Chemother. 2012;56:3898–904. 10.1128/AAC.06120-11 22526317 PMC3393427

[22] Peirano G, Matsumara Y, Nobrega D, DeVinney R, Pitout J. Population-based epidemiology of *Escherichia coli* ST1193 causing blood stream infections in a centralized Canadian region. Eur J Clin Microbiol Infect Dis. 2021. Online ahead of print. 10.1007/s10096-021-04373-5 34750697

[23] Birgy A, Madhi F, Jung C, Levy C, Cointe A, Bidet P, et al.; Group of the National Observatory of Urinary tract Infection due to ESBL-producing Enterobacteriaceae in children. Diversity and trends in population structure of ESBL-producing *Enterobacteriaceae* in febrile urinary tract infections in children in France from 2014 to 2017. J Antimicrob Chemother. 2020;75:96–105. 31617912 10.1093/jac/dkz423

[24] Ding Y, Zhang J, Yao K, Gao W, Wang Y. Molecular characteristics of the new emerging global clone ST1193 among clinical isolates of *Escherichia coli* from neonatal invasive infections in China. Eur J Clin Microbiol Infect Dis. 2021;40:833–40. 10.1007/s10096-020-04079-0 33118058

[25] Zhong Y-M, Zhang X-H, Ma Z, Liu W-E. Prevalence of *Escherichia coli* ST1193 causing intracranial infection in Changsha, China. Trop Med Infect Dis. 2022;7:217. 10.3390/tropicalmed7090217 36136628 PMC9504535

[26] Oldendorff F, Linnér A, Finder M, Eisenlauer P, Kjellberg M, Giske CG, et al. Case report: fatal outcome for a preterm newborn with meningitis caused by extended-spectrum β-lactamase-producing *Escherichia coli* sequence type 1193. Front Pediatr. 2022;10:866762. 10.3389/fped.2022.866762 35463903 PMC9019577

[27] De Francesco MA, Bertelli A, Corbellini S, Scaltriti E, Risso F, Allegri R, et al. Emergence of pandemic clonal lineage sequence types 131 and 69 of extraintestinal *Escherichia coli* as a cause of meningitis: is it time to revise molecular assays? Microbiol Spectr. 2023;11:e0327422. 10.1128/spectrum.03274-22 36786647 PMC10100906

[28] Wyrsch ER, Bushell RN, Marenda MS, Browning GF, Djordjevic SP. Global phylogeny and f virulence plasmid carriage in pandemic *Escherichia coli* ST1193. Microbiol Spectr. 2022;10:e0255422. 10.1128/spectrum.02554-22 36409140 PMC9769970

[29] Johnson TJ, Elnekave E, Miller EA, Munoz-Aguayo J, Flores Figueroa C, Johnston B, et al. Phylogenomic analysis of extraintestinal pathogenic *Escherichia coli* sequence type 1193, an emerging multidrug-resistant clonal group. Antimicrob Agents Chemother. 2018;63:e0191318. 10.1128/AAC.01913-18 30348668 PMC6325179

[30] Jackson N, Belmont CR, Tarlton NJ, Allegretti YH, Adams-Sapper S, Huang YY, et al. Genetic predictive factors for nonsusceptible phenotypes and multidrug resistance in expanded-spectrum cephalosporin-resistant uropathogenic *Escherichia coli* from a multicenter cohort: insights into the phenotypic and genetic basis of coresistance. MSphere. 2022;7:e0047122. 10.1128/msphere.00471-22 36377882 PMC9769571

[31] Hicks LA, Taylor TH Jr, Hunkler RJUS. U.S. outpatient antibiotic prescribing, 2010. N Engl J Med. 2013;368:1461–2. 10.1056/NEJMc1212055 23574140

