# Supplementary material for: Early-Onset Infection Caused by Escherichia coli Sequence Type 1193 in Late Preterm and Full-Term Neonates
Source: Emerg Infect Dis. 2024 Jan;30(1):20–8. doi: 10.3201/eid3001.230851 (PMC10756391; doi:10.3201/eid3001.230851)
Supplement: Appendix — Additional information about early-onset infection caused by Escherichia coli ST1193 in late preterm and full-term neonates. [file 23-0851-Techapp-s1.pdf]

Article DOI: https://doi.org/10.3201/eid3001.230851
EID cannot ensure accessibility for Appendix materials supplied by authors. Readers who have difficulty accessing Appendix content should contact the authors for assistance.

Early-Onset Infection in Late Preterm and Full-Term Neonates Caused by *Escherichia coli* Sequence Type 1193

Appendix

Appendix Table 1. Genes encoding virulence or putative virulence factors among *E. coli* strains in EOS (n = 32) or HVC (n = 50) collections*

Genes†	EOS, n (%)	HVC, n (%)
AslA (Putative sulfatase AslA)	29 (91)	43 (86)
aamR:FN554766	4 (13)	12 (24)
afaA (Putative transcriptional regulator AfaA-VIII)	1(3)	7 (14)
afaB (Chaperone protein AfaB)	1 (3)	6 (12)
afaC (Outer membrane usher protein AfaC)	1 (3)	7 (14)
afaD (aggregative adherence fimbria - Afimbrial adhesion)	2 (6)	10 (20)
afaE (aggregative adherence fimbria - Adhesin protein)	1 (3)	5 (10)
air (Enteroaggregative immunoglobulin repeat protein Air)	6 (19)	10 (20)
anr (Transcriptional activator protein anr)	15 (47)	16 (32)
capU (Hexosyltransferase homologue protein CapU)	7 (22)	12 (24)
Cea (Colicin E1)	8 (25)	16 (32)
chuA (Hemin receptor)	27 (84)	35 (70)
Cia (Colicin Ia)	4 (13)	7 (14)
clbB (Colibactin biosynthesis protein ClbB)	12 (38)	13 (26)
cnf1(Cytotoxic Necrotizing Factor 1 (CNF1))	5 (16)	6 (12)
csgA (Major curlin subunit)	32 (100)	50 (100)
cvaC (Colicin V precursor)	8 (25)	5 (10)
cvi (Colicin V immunity protein)‡	8 (25)	8 (16)
dhaK (dihydroxyacetone kinase)	7 (22)	6 (12)
eilA (Salmonella HilA activator homologue)	6 (19)	11 (22)
espY2:000868321	4 (13)	7 (14)
etsC (Putative type I secretion outer membrane protein EtsC)	8 (25)	9 (18)
fdeC (factor adherence)	32 (100)	45 (90)
fimH (Type 1 fimbrin D-mannose specific adhesin)	31 (97)	48 (96)
focC (S fimbrial/F1C minor subunit)	4 (13)	3 (6)
focCsfaE (S fimbrial/F1C minor subunit)	4 (13)	4 (8)
fyuA (Yersiniabactin siderophore receptor)	31 (97)	42 (84)
Gad (Glutamate decarboxylase (acid resistance))	27 (84)	47 (94)
Hha (Hemolysin expression-modulating protein Hha)	13 (41)	33 (66)
hlyA (Hemolysin A)	7 (22)	8 (16)
HlyE (Hemolysin E)	11 (34)	25 (50)
HlyF (Hemolysin F)	8 (25)	7 (14)
hra (Heat-resistant agglutinin)	6 (19)	12 (24)
ibeA (Invasin IbeA)	7 (22)	6 (12)
Iha (Adherence protein Iha)	17 (53)	27 (54)
Imm (Colicin Ia immunity protein)‡	18 (56)	25 (50)
ireA (Iron-regulated outer membrane virulence protein)	9 (28)	14 (28)
iroN (Salmochelin siderophore receptor IroN)	14 (44)	17 (34)
irp2 (Yersiniabactin siderophore biosynthesis protein Irp2)	31 (97)	42 (84)
iss (Increased serum survival protein)	21 (66)	35 (70)
iucC (Aerobactin siderophore biosynthesis protein IucC)	22 (69)	31 (62)
iutA (Aerobactin siderophore receptor IutA)	22 (69)	31 (62)
kpsE (Capsule polysaccharide export inner membrane protein)	29 (91)	39 (78)
kpsMII (Polysialic acid transport protein)	1 (3)	8 (16)

Genes†	EOS, n (%)	HVC, n (%)
kpsMII_K1 (Polysialic acid transport protein)§	19 (59)	16 (32)
kpsMII_K5 (Polysialic acid transport protein)	5 (16)	9 (18)
mcbA (Bacteriocin microcin B17)	2 (6)	6 (12)
mchB (Microcin H47)	5 (16)	10 (20)
mchC (Microcin H47)	5 (16)	10 (20)
mchF (Transporter protein MchF)	11 (34)	16 (32)
mcmA (Microcin M part of colicin H)	5 (16)	9 (18)
mig14 (Mig-14 protein)‡	7 (22)	5 (10)
neuC (K1 capsule synthesis UDP-N-acetylglucosamine 2-epimerase)§	19 (59)	18 (36)
ompT (Outer membrane protease (protein protease 7)	30 (94)	39(78)
omptp (Outer membrane protease (plasmidic))‡	7 (22)	5 (10)
Pic (Serine protease pic autotransporter)	2 (6)	8 (16)
papA_F43 (Major pilin subunit)	13 (41)	11 (22)
papC (Pyelonephritis associated pili Outer membrane usher protein PapC)	11 (34)	18 (36)
papGII (Pyelonephritis associated pili class II adhesin)‡	7 (22)	13 (26)
sat (Serine protease autotransporters of Enterobacteriaceae (SPATE) Sat)	15 (47)	21(42)
senB (Plasmid-encoded enterotoxin)	17 (53)	19 (38)
sfaD (S fimbrial/F1C minor subunit)	8 (25)	8 (16)
sfaS (S-fimbriae minor subunit)	4 (13)	2 (4)
shiA (Shikimate transporter)	8 (25)	7 (14)
shiB	8 (25)	17 (34)
sitA (Iron transport protein SitA)	31 (97)	41 (82)
tcpC (toll/interleukin-1 receptor domain-containing protein)	4 (13)	6 (12)
terC (Tellurium ion resistance protein)	32 (100)	50 (100)
tia	12 (38)	15 (30)
traJ (Relaxosome protein)	12 (38)	15 (30)
traT (Outer membrane protein complement resistance)	20 (63)	32 (64)
tsh ((SPATE) Temperature-sensitive hemagglutinin)	4 (13)	1 (2)
usp (Uropathogenic-specific protein)	23 (72)	28 (56)
vat ((SPATE) Vacuolating autotransporter toxin)	18 (56)	20 (40)
yehA (Uncharacterized fimbrial-like protein YehA)	31 (97)	49 (98)
yehB (Outer membrane usher protein YehB)	29 (91)	47 (94)
yehC (Probable fimbrial chaperone YehC)	28 (88)	47 (94)
yehD (Uncharacterized fimbrial-like protein YehD)	29 (91)	46 (92)
yfcV (Fimbrial protein)	25 (78)	30 (60)

*EOS, early onset neonatal sepsis; HVC, healthy vaginal carriage
†Only virulence genes with a prevalence of ≥10 in one of the collections are listed.
‡Virulence genes detected by local BLAST (see methods)
§Indicate a significant difference of genes prevalence ($p < 0.05$) between the 2 collections

Appendix Table 2. Genes encoding virulence or putative virulence factors among *E. coli* strains in EOS ST1193 (n = 6) or EOS non–ST1193 (n = 26) collections*

Genes†	ST1193, n (%)	Non–ST1193, n (%)	p-value§
AslA (Putative sulfatase AslA)	6 (100)	23 (88)	>0.05
aamR:FN554766	0 (0)	4 (15)	>0.05
air (Enteroaggregative immunoglobulin repeat protein Air)	0 (0)	6 (23)	>0.05
anr (Transcriptional activator protein anr)	0 (0)	15 (58)	0.02
astA (Arginine N-succinyltransferase)	1 (17)	2 (8)	>0.05
capU (Hexosyltransferase homologue protein CapU)	0 (0)	7 (27)	>0.05
cea (Colicin E1)	0 (0)	8 (31)	>0.05
ccl (cloacin)	1 (17)	1 (4)	>0.05
chuA (Hemin receptor)	6 (100)	21 (81)	>0.05
cia (Colicin Ia)	0 (0)	4 (15)	>0.05
clbB (Colibactin biosynthesis protein ClbB)	1 (17)	11 (42)	>0.05
cnf1 (Cytotoxic Necrotizing Factor 1 (CNF1))	0 (0)	5 (19)	>0.05
colE7 (Colicin-E7)	1 (17)	0 (0)	>0.05
csgA (Major curlin subunit)	6 (100)	26 (100)	>0.05
cvaC (Colicin V precursor)	0 (0)	8 (31)	>0.05
dhaK (dihydroxyacetone kinase)	0 (0)	7 (27)	>0.05
eilA (Salmonella HilA activator homologue)	0 (0)	6 (23)	>0.05
espY2:000868321	0 (0)	4 (15)	>0.05
etsC (Putative type I secretion outer membrane protein EtsC)	0 (0)	8 (31)	>0.05
fdeC (factor adherence)	6 (100)	26 (100)	>0.05
any fimH (Type 1 fimbrin D-mannose specific adhesin)	6 (100)	25 (96)	>0.05
fimH 64 (Type 1 fimbrin D-mannose specific adhesion 64)	6 (100)	0 (0)	<0.001
focC (S fimbrial/F1C minor subunit)	0 (0)	4 (15)	>0.05
focCsfaE (S fimbrial/F1C minor subunit)	0 (0)	4 (15)	>0.05

Genes†	ST1193, n (%)	Non–ST1193, n (%)	p-value§
fyuA (Yersiniabactin siderophore receptor)	6 (100)	25 (96)	>0.05
gad (Glutamate decarboxylase (acid resistance))	6 (100)	21 (81)	>0.05
Hha (Hemolysin expression-modulating protein Hha)	0 (0)	13 (50)	>0.05
hlyA (Hemolysin A)	0 (0)	7 (27)	>0.05
HlyE (Hemolysin E)	0 (0)	11 (42)	>0.05
HlyF (Hemolysin F)	0 (0)	8 (31)	>0.05
hra (Heat-resistant agglutinin)	0 (0)	6 (23)	>0.05
ibeA (Invasin IbeA)	0 (0)	7 (27)	>0.05
Iha (Adherence protein Iha)	6 (100)	11 (42)	0.019
Imm (Colicin Ia immunity protein)**	6 (100)	12 (46)	0.024
ireA (Iron-regulated outer membrane virulence protein)	0 (0)	9 (35)	>0.05
iroN (Salmochelin siderophore receptor IroN)	0 (0)	14 (54)	>0.05
irp2 (Yersiniabactin siderophore biosynthesis protein Irp2)	6 (100)	25 (96)	>0.05
iss (Increased serum survival protein)	0 (0)	21 (81)	<0.001
iucC (Aerobactin siderophore biosynthesis protein IucC)	6 (100)	16 (62)	>0.05
iutA (Aerobactin siderophore receptor IutA)	6 (100)	16 (62)	>0.05
kpsE (Capsule polysaccharide export inner membrane protein)	6 (100)	23 (88)	>0.05
kpsMII_K1 (Polysialic acid transport protein)	6 (100)	13 (50)	>0.05
kpsMII_K5 (Polysialic acid transport protein)	0 (0)	5 (19)	>0.05
mig14 (Mig-14 protein)**	0 (0)	7 (27)	>0.05
mchB (Microcin H47)	0 (0)	5 (19)	>0.05
mchC (Microcin H47)	0 (0)	5 (19)	>0.05
mchF (Transporter protein MchF)	0 (0)	11 (42)	>0.05
mcmA (Microcin M part of colicin H)	0 (0)	5 (19)	>0.05
neuC (K1 capsule synthesis UDP-N-acetylglucosamine 2-epimerase)	6 (100)	13 (50)	>0.05
ompT (Outer membrane protease (protein protease 7)	6 (100)	24 (92)	>0.05
omptp (Outer membrane protease (plasmidic))**	0 (0)	7 (27)	>0.05
papA_F43 (Major pilin subunit)	6 (100)	7 (27)	0.0019
papA_feiA_F8	0 (0)	3 (12)	>0.05
papC (Pyelonephritis associated pili Outer membrane usher protein PapC)	0 (0)	11 (42)	>0.05
papGII (Pyelonephritis associated pili class II adhesin)**	0 (0)	7 (27)	>0.05
sat (Serine protease autotransporters of Enterobacteriaceae (SPATE) Sat)	6 (100)	9 (35)	0.006
senB (Plasmid-encoded enterotoxin)	6 (100)	11 (42)	0.019
sfaD (S fimbrial/F1C minor subunit)	0 (0)	8 (31)	>0.05
sfaS (S-fimbriae minor subunit)	0 (0)	4 (15)	>0.05
shiA (Shikimate transporter)	2 (33)	6 (23)	>0.05
shiB	0 (0)	8 (31)	>0.05
sitA (Iron transport protein SitA)	6 (100)	25 (96)	>0.05
tcpC (toll/interleukin-1 receptor domain-containing protein)	0 (0)	4 (15)	>0.05
terC (Tellurium ion resistance protein)	6 (100)	26 (100)	>0.05
tia	2 (33)	10 (38)	>0.05
• traJ (Relaxosome protein)	0 (0)	12 (46)	>0.05
traT (Outer membrane protein complement resistance)	0 (0)	20 (77)	0.001
Tsh (Temperature-sensitive hemagglutinin)	0 (0)	4 (15)	>0.05
usp (Uropathogenic-specific protein)	6 (100)	17 (65)	>0.05
vat (Vacuolating autotransporter toxin)	6 (100)	12 (46)	0.024
yehA (Uncharacterized fimbrial-like protein YehA)	6 (100)	25 (96)	>0.05
yehB (Outer membrane usher protein YehB)	6 (100)	23 (88)	>0.05
yehC (Probable fimbrial chaperone YehC)	6 (100)	22 (85)	>0.05
yehD (Uncharacterized fimbrial-like protein YehD)	6 (100)	23 (88)	>0.05
yfcV (Fimbrial protein)	6 (100)	19 (73)	>0.05

*ST, sequence type
†Only virulence genes with a prevalence of ≥10 in one of the collections are listed.
‡Virulence genes detected by local BLAST
§p value calculated with Fisher exact analysis, threshold for significance: <0.05.

Appendix Table 3. Healthy vaginal colonization *E. coli*

Isolates	N50	Coverage
21660147MF_S24	244667	169
21670134KC_S29	59409	79
21680051BM_S28	108785	96
21680077DG_S25	355528	122
21680084BFK_S27	104814	95
21680097DM_S26	245687	152
21710028CP_S30	177894	79
21710041BE_S31	300298	113
21710098-BB_S20	219523	65
21720121-SN_S21	117586	67
21730098-SS_S22	101344	68
21740131-BS_S23	217515	95
21820063-RMH_S25	106321	57
21870051-DM_S24	59348	67
21960106-DK_S26	108429	91
21990114-RA_S27	57564	106
22010042-BA_S28	275248	109
22010109-WA_S29	240556	49
AVC1RV_S1	263633	93
AVC2MJ_S2	127932	125
AVC3BA_S3	177581	147
AVC4CR_S4	127841	110
AVC5LA_S5	228082	121
AVC6VE_S6	86987	68
AVC7AK_S7	287923	153
AVC8DD_S8	111095	114
AVC-9-a.m._S30	259573	85
AVC11GA_S10	203351	86
LMR1EL_S11	191062	99
LMR2CK_S12	544568	106
LMR4DM_S14	77856	34
LMR6MC_S16	388705	57
LMR7BD_S17	95117	120
LMR8MR_S18	212470	103
LMR9PO_S19	122371	82
LMR10AS_S20	58328	113
AVC-10-CM	203897	136
21540126-BH	259845	140
21610097-KH	261749	164
LMR3-BL	93255	141
LMR5-ES	60504	118
LRB1	94759	117
LRB3	305009	200
LRB4	377622	188
LRB5	191824	97
LRB6	179883	128
LRB7	240768	113
LRB8	99769	116
LRB10	269065	87
LRB11	78259	88
Maximum	544568	200
Minimum	57564	34
Median	185472.5	106

Appendix Table 4. Early onset neonatal sepsis *E. coli*

Isolates	N50	Coverage
APIMF52	333512	154
APIMF53	298766	140
APIMF54	79272	90
APIMF56	168482	133
APIMF57	109201	159
APIMF58	246576	66
APIMF59	223171	126
APIMF60	210541	114
APIMF63	104989	106
APIMF67	341395	152
APIMF68	178169	78
APIMF69	419368	74
APIMF70	66787	102
APIMF71	551760	117
APIMF72	563762	88
APIMF73	215239	114
APIMF76	210541	71
APIMF77	204129	85
APIMF78	274362	99
APIMF79	171995	86
APIMF80	211242	90
APIMF81	196353	89
APIMF82	237021	68
APIMF83	134788	106
APIMF84	223226	85
APIMF85	341964	55
APIMF87	94828	73
APIMF88	111031	86
APIMF89	209517	97
APIMF90	216134	88
APIMF91	61362	74
APIMF92	204007	130
Maximum	563762	159
Minimum	61362	55
Median	210541	90
